# Pixantrone induces cell death through mitotic perturbations and subsequent aberrant cell divisions

**DOI:** 10.1080/15384047.2015.1070979

**Published:** 2015-07-15

**Authors:** Neil Beeharry, Andrea Ghelli Luserna Di Rora, Mitchell R Smith, Timothy J Yen

**Affiliations:** 1 Cancer Biology Program; Fox Chase Cancer Center; Philadelphia, PA USA; 2 LAM Therapeutics; Guilford, CT USA; 3 Dip. di Medicina Specialistica Diagnostica e Sperimentale; Università di Bologna; Bologna, Italy; 4 Department of Hematology and Oncology; Taussig Cancer Institute; Cleveland Clinic; Cleveland, OH USA

**Keywords:** cell cycle, cell division, chromosome instability, DNA damage, mitotic catastrophe

## Abstract

Pixantrone is a novel aza-anthracenedione active against aggressive lymphoma and is being evaluated for use against various hematologic and solid tumors. The drug is an analog of mitoxantrone, but displays less cardiotoxicity than mitoxantrone or the more commonly used doxorubicin. Although pixantrone is purported to inhibit topoisomerase II activity and intercalate with DNA, exact mechanisms of how it induces cell death remain obscure. Here we evaluated the effect of pixantrone on a panel of solid tumor cell lines to understand its mechanism of cell killing. Initial experiments with pixantrone showed an apparent discrepancy between its anti-proliferative effects in MTS assays (short-term) compared with clonogenic assays (long-term). Using live cell videomicroscopy to track the fates of cells, we found that cells treated with pixantrone underwent multiple rounds of aberrant cell division before eventually dying after approximately 5 d post-treatment. Cells underwent abnormal mitosis in which chromosome segregation was impaired, generating chromatin bridges between cells or within cells containing micronuclei. While pixantrone-treated cells did not display γH2AX foci, a marker of DNA damage, in the main nuclei, such foci were often detected in the micronuclei. Using DNA content analysis, we found that pixantrone concentrations that induced cell death in a clonogenic assay did not impede cell cycle progression, further supporting the lack of canonical DNA damage signaling. These findings suggest pixantrone induces a latent type of DNA damage that impairs the fidelity of mitosis, without triggering DNA damage response or mitotic checkpoint activation, but is lethal after successive rounds of aberrant division.

## Abbreviations

DOX: doxorubicin
Gem: gemcitabine
kT: kinetochores
MTS: 3-(4,5-dimethylthiazol-2-yl)-5-(3-carboxymethoxyphenyl)-2-(4-sulfophenyl-2H-tetrazolium
NHL: Non-Hodgkin's lymphoma
PIX: pixantrone
RPA: replication protein A
TOPO II: topoisomerase II.

## Introduction

Pixantrone (PIX) is an aza-anthracendione with cytotoxic activity against a variety of cancer cell lines. The drug is particularly efficacious in hematologic cancers, having been approved in Europe for use in adult patients with relapsed or refractory aggressive non-Hodgkin's B-cell lymphoma (NHL).^1,2^ A critical observation from several *in vivo* studies is that the cardiotoxicity associated with doxorubicin was not detected in animals treated with pixantrone. Moreover, recent biochemical studies in human cardiac myocytes demonstrated that PIX does not generate reactive oxygen species, probably due to its inability to interact with mitochondrial iron.^3,4^ Despite the favorable preclinical and clinical findings regarding both efficacy and toxicity, a definitive mechanism of action for PIX-induced cell killing is still lacking.

*In vitro* studies have established that PIX can affect DNA topology through a number of mechanisms. First, PIX interacts with topoisomerase II (TOPO II), a nuclear enzyme that regulates DNA topology and is considered to be an important target given the clinical efficacy of doxorubicin and etoposide.^5^ Inhibition of TOPO II traps and stabilizes the transient protein-DNA complex, resulting in the generation of double strand breaks and eventual cell death (For a review see ref.^6^). PIX, however, is a much weaker inhibitor of TOPO II, than the structurally related drug mitoxantrone or doxorubicin, suggesting this may not be the major mechanism for inducing cell death. Further, the cytotoxic activity of anthracenediones does not clearly correlate with their ability to induce double strand breaks.^7^ Second, NMR spectroscopic studies showed that PIX intercalates into DNA.^8^ Finally, a mechanism dependent upon formaldehyde to generate covalent drug-DNA adducts has been described.^9^ Taken together, these studies establish that DNA is a *bona fide* target of PIX, be it directly or indirectly. What remains more difficult to assess is how this interaction with DNA manifests in the cytotoxic action of PIX and confers non-cross-resistance with anthracyclines.

Perturbation of cell cycle dynamics commonly occurs in cells treated with DNA interacting agents. The activation of a complex series of biochemical reactions ultimately prevents cells from entering mitosis with damaged DNA, thereby maintaining genomic stability. Thus, cell cycle checkpoints serve as sentinel mechanisms that are critical to ensure cell viability. Cell cycle checkpoint activation is tightly coupled with DNA repair. Thus, if the DNA damage is successfully repaired, cell cycle arrest is alleviated and cell cycle progression is resumed. However, sustained DNA damage will eventually result in cell death.^10^

In this report, the effect of PIX is examined on a number of solid tumor cell lines. At concentrations that reduced clonogenic cell survival, there was no detectable DNA damage induction. However, we found that PIX affected chromosome dynamics in mitosis resulting in the generation of lagging chromosomes and micronuclei. Using live-cell videomicroscopy we demonstrate that cells are able to undergo several rounds of abnormal mitosis before eventually dying. These findings describe a previously unreported mechanism of action of PIX-induced cell death.

## Results

### Pixantrone reduces proliferation in multiple cancer cell lines independent of cell cycle perturbation

**Figure 1.**
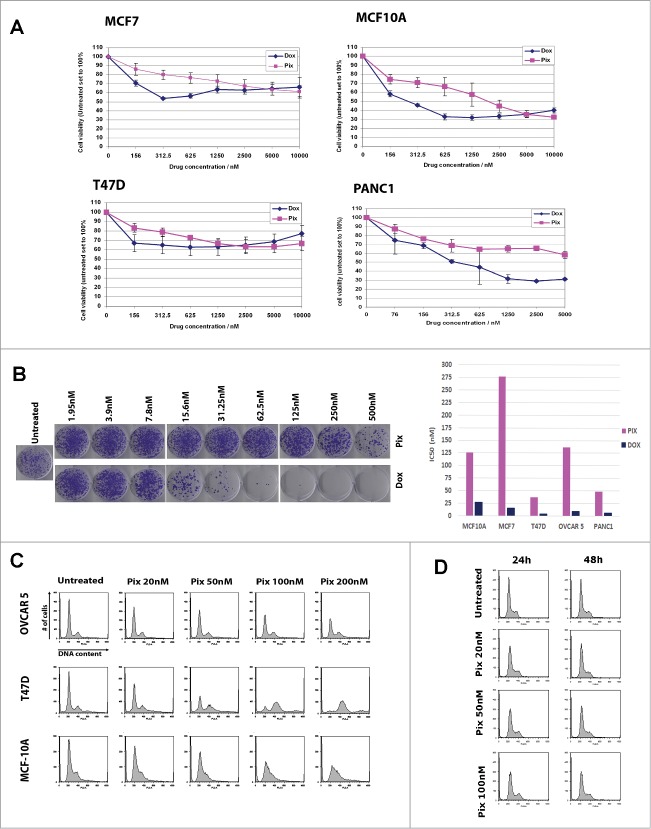
Pixantrone induces cell death in multiple cancer cell lines independent of cell cycle perturbation. (**A**) MTS assays: MCF7, MCF-10A, T47D and PANC1 cells were seeded into 96-well plates, treated with increasing concentrations of pixantrone (pink lines) or doxorubicin (dark blue lines) for 72 hours. Cell viability was determined using MTS reagent. Cells were seeded in triplicate and conducted a minimum of 3 times. Data shown are relative to untreated control cells (set to 100%) ± SD. (**B**) Clonogenic assays: Clonogenic survival assays were performed on indicated cell lines. Cells were treated with indicated concentrations of pixantrone (Pix) or doxorubicin (Dox) for 24h after which cells were grown in drug-free medium for an additional 9 d A representative clonogenic assay using MCF-7 cells is shown in the left panel. Solubilization of crystal violet stained colonies was used to quantify clonogenicity. Cells were tested in duplicate, at least twice. Data presented are the IC_50_ values of either pixantrone or doxorubicin in the indicated cell line (right). (**C**) Representative cell cycle profiles are shown for of OVCAR5, T47D, and MCF-10A cells treated with increasing concentrations of pixantrone for 24 hours. (**D**) Representative cell cycle profiles are shown of PANC1 cells treated with increasing concentrations of pixantrone for 24 hours or 48 hours.

The effects of PIX on cell proliferation were tested against a variety of solid tumor cell lines. Breast cancer cell lines (MCF7, T47D and MCF10A; non-transformed breast epithelial cells), pancreatic adenocarcinoma (PANC1) and ovarian cancer cell lines (OVCAR 5, OVCAR 10 and PEO1) were treated for 72 hours with PIX or doxorubicin (DOX). The results showed that PIX did not greatly affect proliferation in the short-term cell viability assay (**Fig. 1A** and data not shown). The clonogenic assay was used to better simulate the *in vivo* setting - chronic treatment followed by a drug-free period. Thus, cells were treated with different concentrations of PIX for 24 hours, followed by drug washout and then incubation for 9 d in the absence of drug. After this period, surviving colonies were fixed, stained, and quantified. Under these conditions, we found that PIX dose-dependently reduced colony formation in all cell lines tested (**Fig. 1B** and Supp. **Fig. 1**). Using the same method, it was observed that cells were 4.5–18.5 times more sensitive to DOX (**Fig. 1B** and Supp. **Fig. 1**).

Next, we observed that PIX concentrations that reduced clonogenic survival also impacted cell cycle dynamics. T47D cells that were the most sensitive to PIX in proliferation assays (IC_50_ = 37.3 nM) were also the most sensitive in cell cycle studies, as evidenced by reduced G1 fraction at 50 nM and increased G2 / M fraction above 100 nM. We also noted the presence of a population of cells with >4N DNA. While MCF-10A and OVCAR5 cells had an intermediate sensitivity to PIX (IC50 = 126 nM and 136 nM, respectively), their cell cycle profiles were distinct. MCF10A cells underwent a G1-mediated arrest at concentrations >100nM, while OVCAR5 cells displayed minimal cell cycle perturbation at 200 nM (**Fig. 1C** and Supp. **Fig. 2**). Finally, PANC1 cells underwent no obvious changes in cell cycle dynamics when treated with PIX at 100 nM, a concentration 2-fold greater than the IC_50_, for 24 or 48 hours (**Fig. 1D**). Taken together, these results suggest that the PIX-mediated reduction in clonogenic survival is not fully explained by PIX's ability to impair cell cycle progression.

### Pixantrone induces DNA damage only at concentrations above cytotoxic levels

**Figure 2.**
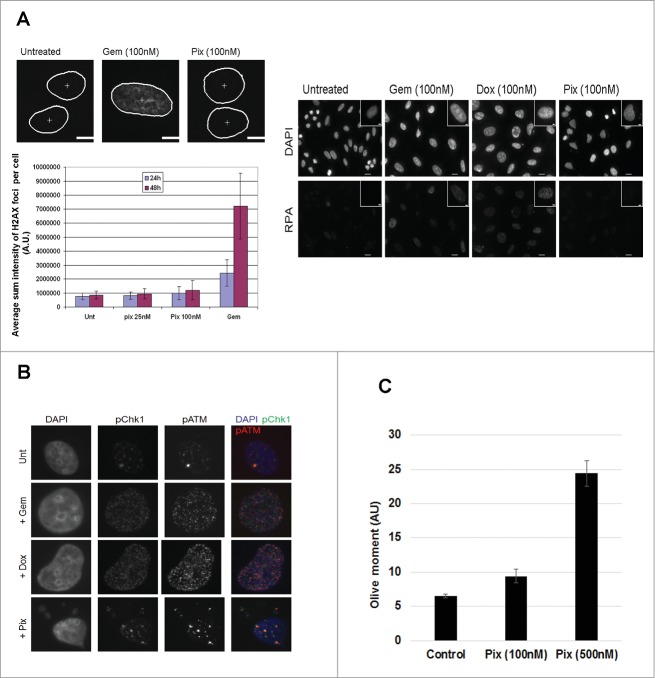
Pixantrone induces DNA damage at high concentrations but not at concentrations sufficient to kill cells. (**A**) PANC1 cells were treated with pixantrone (25 or 100 nM) or gemcitabine (100 nM) for 24 hours or 48 hours. Cells were then fixed and immuno-stained for γH2AX. Representative images, which were used for quantitative image analysis, are shown with the nuclei (white outline) and γH2AX staining. Quantification of γH2AX signal per cell ± standard error of the mean (SEM) was peformed from a minimum of 100 cells (lower left). Cells treated with gemcitabine (Gem, 100 nM), doxorubicin (Dox, 100 nM) or pixantrone (Pix, 100 nM) for 24 hours were fixed and immune-stained for replication protein A (RPA) and counter-stained with DAPI. Representative images are shown. (**B**) PANC1 cells were treated with Gem (100 nM), Dox (100 nM) or Pix (100 nM) for 24 hours. Coverslips were fixed and immuno-stained with pChk1 (green) or pATM (red) and counter-stained with DAPI (blue). Representative cells are shown. (**C**) The alkaline Comet assay was conducted on PANC1 cells treated with pixantrone at 100 or 500 nM for 24 hours. Studies were performed twice, with a minimum of 70 cells being scored for each experiment. Data presented are the Olive moment ± SEM. * P< 0.0001 compared to control cells.

Previous studies have shown that PIX intercalates into DNA,^7^ so a study was performed to test whether PIX induces DNA damage at the concentrations that reduced colony survival (as observed in **Fig. 1B**). Immunofluorescence staining was used to detect the accumulation of various DNA damage response proteins that appear as discrete foci at sites of damage. Indeed, when PANC1 cells were treated with gemcitabine, an anti-metabolite that blocks DNA replication and causes cells to arrest in S phase, there was an increase in foci formation of γ-H2AX and Replication Protein A (RPA), as expected.^11,12^ By contrast, PIX at a concentration that reduced clonogenic survival by ∼75% (100 nM) did not induce γ-H2AX or of RPA foci formation, suggesting no DNA breaks were present (**Fig. 2A**). Interestingly, p-ATM, pChk1 and p-Chk2 formed discrete foci at this concentration of PIX, but the foci were larger than those formed by DOX or gemcitabine treatment (**Fig. 2B** and data not shown). However, when PANC1 cells were treated with PIX (500 nM), the presence of γ-H2AX and 53BP1 foci were clearly detected (data not shown). These findings indicated that PIX did not induce canonical DNA damage at concentrations sufficient for cell killing (100 nM), although higher concentrations (500 nM) induced extensive DNA damage. To directly test this supposition, DNA breaks were measured using the comet assay using cells treated with PIX at 100 or 500 nM. As shown in **Figure 2C**, cells treated with PIX at 100 nM did not significantly increase DNA damage (Control Olive moment 6.5 ± 0.3 vs. 9.4 ± 1.0, NS). However, cells treated with PIX at 500nM had increased DNA damage (Olive moment 24.4 ± 1.9; P < 0.0001), consistent with the immunofluorescence data.

### Pixantrone induces severe chromosomal aberrations and mitotic catastrophe

**Figure 3.**
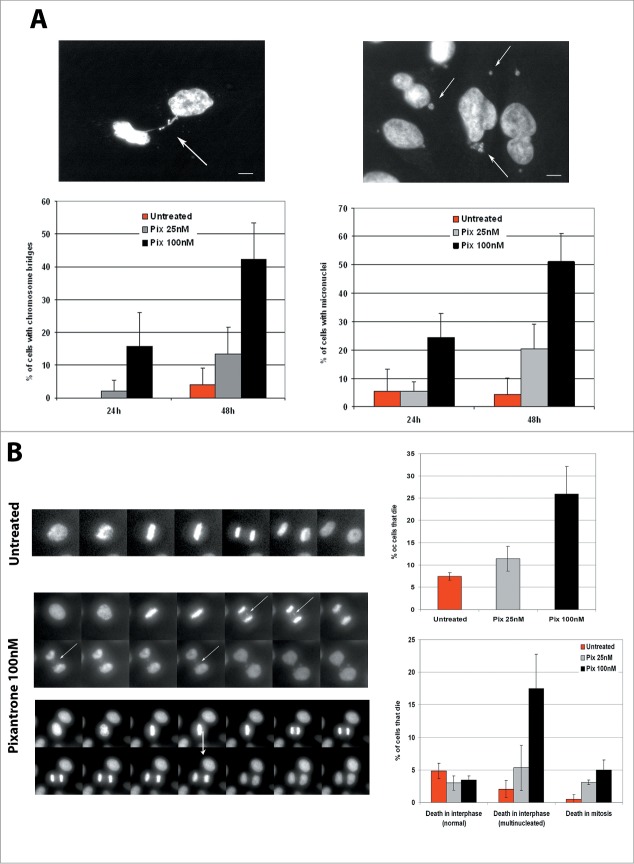
Pixantrone induces severe chromosomal aberrations and mitotic catastrophe. (**A**) PANC1 cells were treated with pixantrone (25 or 100 nM) for 24 hours or 48 hours before cells were fixed and stained with DAPI to ascertain chromosomal aberrations. The percentage of cells displaying chromosomal bridges (left) or micronuclei (right) was quantified. Two independent studies were performed, each scoring a minimum of 300 cells per condition. Data shown is the mean +/− SEM. (**B**) PANC1 cells stably expressing histone H2B: GFP were used for live-cell videomicroscopy. Cells were treated with PIX (100nM) just prior to starting filming. Left side shows representative montages from untreated and PIX treated cells undergoing cell division. The arrows denotes cells exhibiting chromosomal bridges that formed during mitosis. Right side shows the quantification of cells that die during filming (upper) and when cell death happened (lower). Each frame represents 5 minute intervals. Movies were performed twice and a minimum of 100 cells per condition were scored.

Based on these findings, we reasoned that cell death induced by PIX did not occur through a DNA damage-mediated mechanism but must affect another key biological process. Corroborating this notion were observations from immunofluorescence images of PANC1 cells treated with PIX (100 nM) which occasionally showed various chromosomal aberrations. Extending this observation, we tested if PIX could affect chromosome segregation in mitosis. We examined dividing cells for the presence of chromatin bridges as an indicator of mitotic fidelity. Quantitative analyses revealed a concentration and time dependent increase in the number of chromatin bridges formed after PIX treatment (**Fig. 3A**). Chromatin bridges were detected only after 48 hours of continuous treatment with 25 nM PIX (at 24 hours: untreated cells 0 versus PIX 25 nM 2.0% ± 3.3% of cells; at 48 hours: untreated cells 4.0% ± 5.0% vs. PIX 25 nM 13.3% ± 8.1% of cells). However, an increase in bridge formation was evident after 24 hours treatment with 100 nM PIX (15.7% ± 10.5 % of cells). In addition to chromatin bridge formation, an increase was noted in micronucleated cells after PIX treatment. This was evident 24 hours post-treatment with 100 nM PIX or 48 hours post-treatment with 25 nM PIX (**Fig. 3A**). These findings suggested that the formation of chromatin bridges, and subsequent breakage during division likely led to the generation of micronuclei. To directly assess this, live-cell video microscopy was using on PANC1 cells stably expressing histone H2B: GFP to track chromosome dynamics in response to PIX treatment. Vehicle-treated cells progressed normally through mitosis to produce 2 normal daughter cells. However, cells treated with PIX often displayed lagging chromosomes that were the source of chromatin bridges and micronuclei (**Fig. 3B**). Pertinently, cells with these chromosomal aberrations continued to cycle and continued to divide with normal cell cycle timing, and they eventually produced micronucleated cells. These abnormal cells eventually died in interphase after approximately 3 divisions (death in interphase (multinucleated)) (**Fig. 3B**). These findings provide a mechanistic explanation for the apparent lack of toxicity measured in the short term MTS assay as compared with the longer-term clonogenic assay.

To test whether the generation of chromatin bridges resulted from the effects of PIX on processes prior to or during mitosis, PIX was directly added to cells in mitosis. No increase was seen in the frequency of chromatin bridges compared with untreated cells. This suggested that the chromatin bridges resulted from perturbation of events during interphase. Therefore, PIX was added to cells at 2 (early S phase) and 6 hours (late S phase) after release from a thymidine induced G1/S arrest. Cells were allowed to proceed through mitosis before they were fixed and stained. Regardless of the amount of time that these cells were exposed to PIX in S phase, they exhibited a 2-fold increase in the frequency of bridges relative to untreated cells.

### Pixantrone may disrupt chromosome segregation because it generates merotelic kinetochore attachments that lead to chromosome non-disjunction

**Figure 4.**
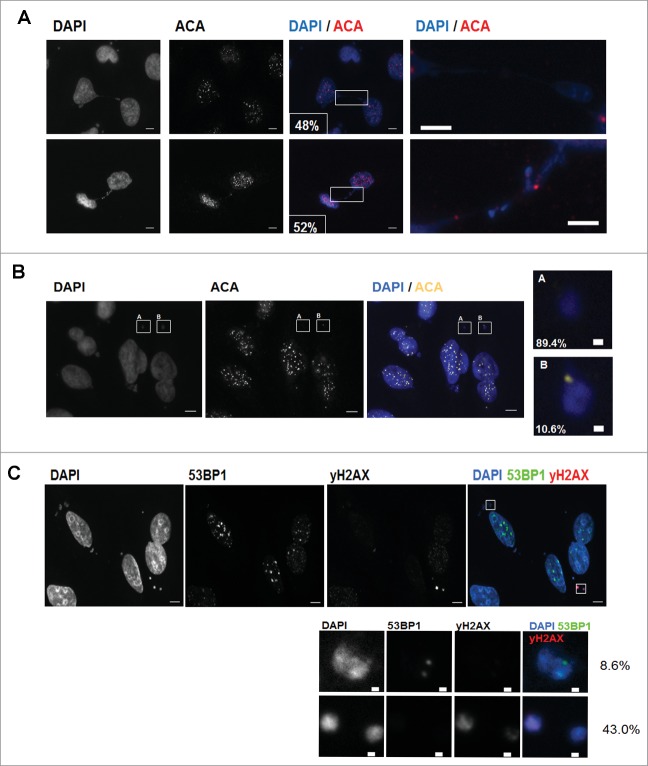
Pixantrone may disrupt chromosome segregation because it generates merotelic kinetochore attachments that lead to chromosome non-disjunction. PANC1 cells treated with pixantrone (100 nM) for 24hours were fixed for immunostaining. (**A**) Antibodies ACA (red) was used to detect centromeres while DAPI (blue) was used to detect DNA. The percentage of cells that displayed chromosome bridges that contained centromeres were quantified. A minimum of 100 cells were scored. (**B**) ACA-negative (inset A) or ACA positive (inset B) micronuclei were quantified from a minimum of 100 cells. (**C**) Drug-treated cells were immunostained with 53BP1 (green) or γH2AX (red) and counterstained with DAPI (blue). The percentage of micronuclei that contained 53BP1 or γH2AX foci were quantified. A minimum of 100 cells containing micronuclei were scored. Insets show magnified view of selected micronuclei.

The centromeres of chromosomes were tracked after PIX treatment, because doing so could provide clues about chromosome missegregation. PANC1 cells were treated with PIX at 100 nM overnight and stained with an anti-centromere antibody (ACA-autoimmune serum that recognizes CENP-A, B and C proteins). Inspection of cells with chromosome bridges showed that approximately 50% of the bridges exhibited paired foci of centromere staining (each pair represents proteins localized at the 2 sister kinetochores (kT) that occupy the 2 sides of the primary constriction) (**Fig. 4A**). Therefore, these chromosomes failed to properly segregate, as with other chromosomes in the cell. The lagging chromosomes eventually formed micronuclei that were separated from the nucleus. Micronuclei that arise from non-disjunction differ from those that form from random pieces of broken chromosomes because they retain their centromeres. Approximately 10% of the micronuclei that were treated with PIX were ACA-positive (**Fig. 4B**). Moreover, by staining for γ-H2AX and 53BP1, DNA damage was observed in the micronuclei 51.6% (43% for γ-H2AX, 8.6% for 53BP1) of the time (**Fig. 4C**).

## Discussion

PIX has demonstrable clinical anti-tumor activity, with less cardiotoxicity than mitoxantrone or DOX. PIX is structurally similar to mitoxantrone and has been clinically considered similar to DOX, but it is important to understand its difference from these agents and its precise mechanism of action. In fact, despite DNA being a known target of PIX, be it through direct intercalation or through affecting its topology via inhibition of TOPO II - questions still remain about how PIX induces cell death. In this study, the effects of PIX were examined on a panel of solid tumor lines. We found that PIX was less potent in cell viability than doxorubicin at equimolar concentrations, both in short-term and in longer-term assays, consistent with previous *in vitro* and *in vivo* data.^13^ Interestingly, the cell line (T47D) most sensitive to doxorubicin was also most sensitive to PIX. This finding suggests that PIX is efficacious in doxorubicin-sensitive cell lines and may be considered in regimens that use doxorubicin.

Previous studies have demonstrated that PIX can influence DNA biology in both an indirect and/or a direct manner. Indeed, it has been shown that intercalation of PIX into DNA likely contributes to its cytotoxic action.^8^ Moreover, by studying mitoxantrone and a variety of structurally related 2-aza derivatives, De Isabella et al. demonstrated that compounds that inhibit TOPO II still retain significant cytotoxic activity.^14^ As shown here, limited potency was observed in a standard 3 day assay, but PIX did reduce proliferation after chronic (24 h) exposure followed by 9 d of drug-free conditions, suggesting a temporal component to PIX-mediated cell death. When further examined at concentrations that reduced clonogenic survival, little or no effects were seen on DNA damage, as ascertained by IF and comet assay. Consistently, no effects on cell cycle were observed at these concentrations.

We previously demonstrated that sustained DNA damage by bendamustine, a chemotherapeutic agent, results in sustained cell cycle arrest and eventual cell death.^10^ The studies presented here show that PIX, at concentrations that reduce clonogenic survival, does not induce DNA damage. This was evidenced by lack of γ-H2AX/RPA foci and also no increase in Olive moment using the comet assay. However, a 5-fold increase in PIX concentration did induce both γH2AX foci and increased the Olive moment, suggesting PIX was able to induce DNA damage, although exceeding the concentration that is sufficient to be cytotoxic. This observation is consistent with another study that determined DNA damage via the comet assay.^15^ Using MDA-MB-231 cells treated with a wide range of PIX (0 to 2000 nM), the authors only detected a significant increase in the Olive tail moment at concentrations at and above 500 nM. The apparent discrepancy was noted about PIX's ability to significantly reduce clonogenic survival, yet not induce DNA damage (at 100 nM). Our studies using live-cell video-microscopy demonstrated that PIX-treated cells were able to enter and exit mitosis. However, there was an increase in chromosome bridges during mitosis and this observation is strongly indicative of merotelic kinetochore attachments, a defect whereby a sister kinetochore is attached to microtubules from both poles (as opposed to the normal case where each sister kT attaches to microtubules from only one pole) (For a review see ref.^16^). These defective attachments are not sensed by the mitotic checkpoint, which explains why PIX treated cells were not delayed in mitosis (timelapse studies not shown). Once in anaphase, the merotelically attached chromosomes are unable to be pulled to opposite poles because of the aberrant geometry of the attachments, and thus remained between the dividing cell. This is supported by the fact that 50% of the lagging chromosomes exhibit paired sister kinetochores. Over time, one of the aberrant attachments gives way and the whole chromosome is pulled to one daughter cell that results in non-disjunction.^16^ Other outcomes may also include chromosome breakage.

PIX did not induce bridges when added to mitotic cells; therefore, the drug had to be present during interphase to generate chromosome bridges. This suggests that the mitotic defects resulted from perturbation of some interphase event(s) that are critical for centromere and kinetochore functions. Interestingly, the occurrence of these bridges did not result in immediate cell death but rather generated micronuclei when chromosomes decondensed during mitotic exit. The generation of PIX-induced micronuclei has 2 important implications. First, studies by Utani et al. found that cells with micronuclei frequently generated daughter cells with additional micronuclei leading to highly multinucleated cells. The generation of such highly multinucleated cells compromised cell viability and ultimately led to apoptotic cell death.^17^ Second, the identification of micronuclei has been successfully used as a biomarker to assess the efficacy of cancer radiotherapy (For a review see ref.^18^). Thus, the identification of micronuclei may similarly serve as a useful biomarker of PIX efficacy.

An unresolved observation from this study is that while no increase in DNA damage was detected, as evidenced by performing standard assays to identify γ-H2AX or 53BP1 foci or via the comet assay, other proteins relating to DNA damage and repair, namely - p-ATM, p-Chk1 and pChk2 – were present. However, the presence of such proteins following PIX treatment did not appear to induce cell cycle delays. Studies are underway to further investigate this observation. An interesting observation by Ichijima et al. may shed light on the mechanism.^19^ These authors showed that forcing cells through the cell cycle via E2F over-expression induced DNA replication stress. Critically, this was characterized by a lack of γ-H2AX foci, but the presence of a few, large p-ATM foci and also increased frequency of chromosome bridges. These observations are reminiscent of the findings in the present study regarding cells treated with PIX and stained for p-ATM as well as p-Chk1 and p-Chk2 (see **Fig. 2**), and the increase in chromosome bridges. This study showed that this occurred in cells treated with PIX in interphase (S / G2), but not mitosis; thus, these data agree with the conclusions of Ichijima et al, that the limited number or type of DNA lesions generated may not be sufficient to trigger a checkpoint response but can have delayed effects when carried into mitosis.^19^

The concentrations used in this study are well within a clinically achievable mean plasma concentrations, which are approximately 4 µM.^20^ Our data suggests that PIX-induced cell death does not occur via intercalation per se, but is the result of successive rounds of aberrant mitoses prior to cell death. Thus, PIX-induced cell death is dependent upon cell division. This has implications for rational combinations of agents to be developed, and for the importance of investigation sequence of administration.

## Methods and Materials

### Reagents

The following reagents were obtained from Sigma: doxorubicin and propidium iodide. Gemcitabine was a commercial supply (obtained from the FCCC pharmacy). Pixantrone was kindly provided by CTI BioPharma Corp.

### Cell Culture

Cell lines were obtained from ATCC and banked at Fox Chase Cancer Center (FCCC, Philadelphia, PA USA) until use. Mycoplasma testing was conducted at FCCC prior to these studies. MCF7, MCF-10A and OVCAR-5, -10 and PEO1 cells were grown in RMPI-1640, while PANC1 cells were grown in DMEM. All media was supplemented with 10% FBS, 2 mM glutamine, 1% penicillin, and streptomycin.

### Cell Proliferation Assays

Cell proliferation was performed as previously described.^10^ Briefly, cells seeded into 96-well plates were treated with increasing concentrations of either pixantrone or doxorubicin for 72 hours. After this time, MTS reagent (Promega) was added to cells and incubated at 37°C for a further 4 hours according to the manufacturer's protocol. Cell proliferation was then determined by measuring the absorbance at 490nm. All data points were normalized to untreated cells. All treatments were performed in triplicate and performed a minimum of 3 times.

For clonogenic assays, 1000 cells per well were seeded into a 6-well plate. Cells were treated with PIX or doxorubicin at the indicated concentrations for 24 hours. Following this time, drugs were washed out and cells were cultured in drug-free medium for an additional 9 d before being fixed and stained with crystal violet for quantitation as previously described.^10^ The IC_50_ values were determined using GraphPad Prism 4® software.

### Microscopy

For time-lapse studies, PANC1 cells stably expressing GFP-histone H2B were used as previously described.^10^ Briefly, cells were seeded into 12-well plates and incubated for 24 hours. Pixantrone at various concentrations was then added to cells immediately before time-lapse videomicroscopy was commenced. The multi-well plate was placed into a heated chamber and bright-field and fluorescent images were taken every 5 minutes for up to 48 hours, using a Nikon TE2000S microscope (Nikon) controlled by Metamorph (Molecular Devices). To quantify the fates of cells for each condition, movies were examined frame by frame, and at least 100 cells were counted, from a 2 independent movies. Selected frames were chosen for montages to highlight cells undergoing aberrant mitosis.

### Immunofluorescence

For immunofluorescence, cells were seeded onto coverslips 24 hours prior to drug treatment. Cells were synchronized with thymidine prior to drug treatments as described above. Six to 9 hours after addition of checkpoint inhibitors, cells were fixed (4% paraformaldehyde/PBS) and stained as previously described.^21^ Antibodies to ACA (a gift from Dr. JB Rattner, University of Alberta), 53BP1 (Abcam) and γH2AX (Upstate) were used. Alexa Fluor-conjugated secondary antibodies (488, 555, 647nm) were used at a final concentration of 1 μg/ml (Invitrogen) and cells were counterstained with DAPI (Molecular Probes). Images were captured using a 40× or 100× objective mounted on an inverted microscope (Eclipse TE2000S; Nikon) with a charge-coupled device camera (Photometrics Cascade 512F; Roper Scientific) using Nikon Elements 2.0 (Nikon).

### Comet assay

PANC1 cells were treated with PIX at 25, 100 or 500 nM for 24 hours. Drug-treated cells were processed and analyzed as previously described.^21^ A minimum of 70 cells were scored and the assays were performed 2 independent times. Images were acquired as previously described^21^ and analyzed using TriTek CometScore™ software. The Olive moment was used to define the extent of DNA damage.

## Supplemental Material

Supplemental Figures and Captions
