# Supplementary material for: Pixantrone induces cell death through mitotic perturbations and subsequent aberrant cell divisions
Source: Cancer Biol Ther. 2015 Jul 15;16(9):1397–406. doi: 10.1080/15384047.2015.1070979 (PMC4621998; doi:10.1080/15384047.2015.1070979)
Supplement: Supplemental Figures and Captions [file kcbt-16-09-1070979-s001.zip › 1070979 Supplemental Figure Captions.docx]

**Supplemental Figures**

**Supplemental Figure 1**

Clonogenic assays were performed in **A.** OVCAR-5 and **B.** PANC1 cells treated with pixantrone (PIX) or doxorubicin (Dox) at the indicated concentrations. 24h after drug treatment, drugs were washed out and cells were cultured for an additional 10 days. Representative clonogenic assays are shown. Studies were performed in duplicate and conducted two independent times.

**Supplemental Figure 2**

The cell cycle profiles from those shown in figure 1C were quantified with respect to the percentage of cells in G1, S or G2 phases after treatment with pixantrone (20 – 200 nM) for 24h.
