# Supplementary figures and images for: Pixantrone induces cell death through mitotic perturbations and subsequent aberrant cell divisions
Source: Cancer Biol Ther. 2015 Jul 15;16(9):1397–406. doi: 10.1080/15384047.2015.1070979 (PMC4621998; doi:10.1080/15384047.2015.1070979)

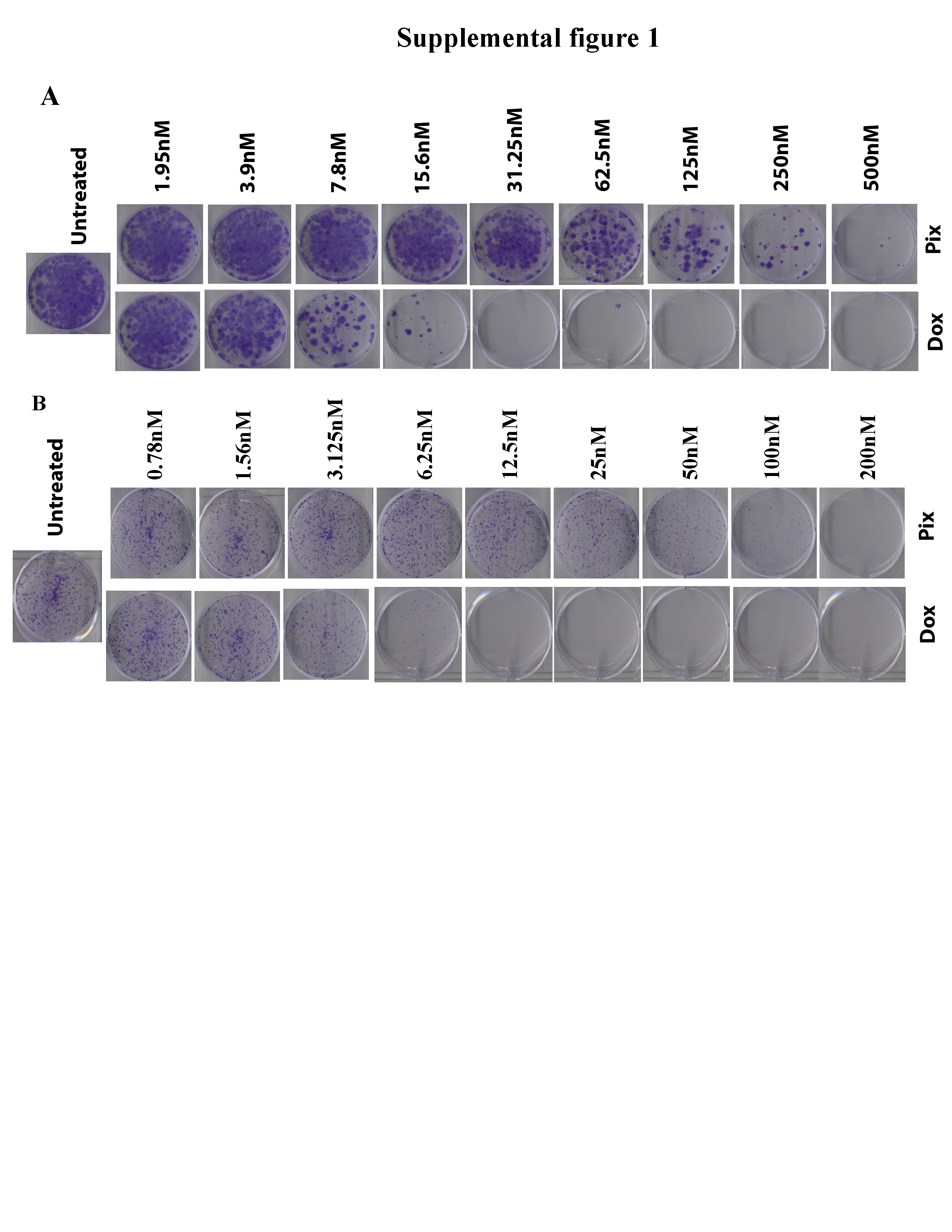

Supplement: Supplemental Figures and Captions [file kcbt-16-09-1070979-s001.zip › Figure S1.jpg]

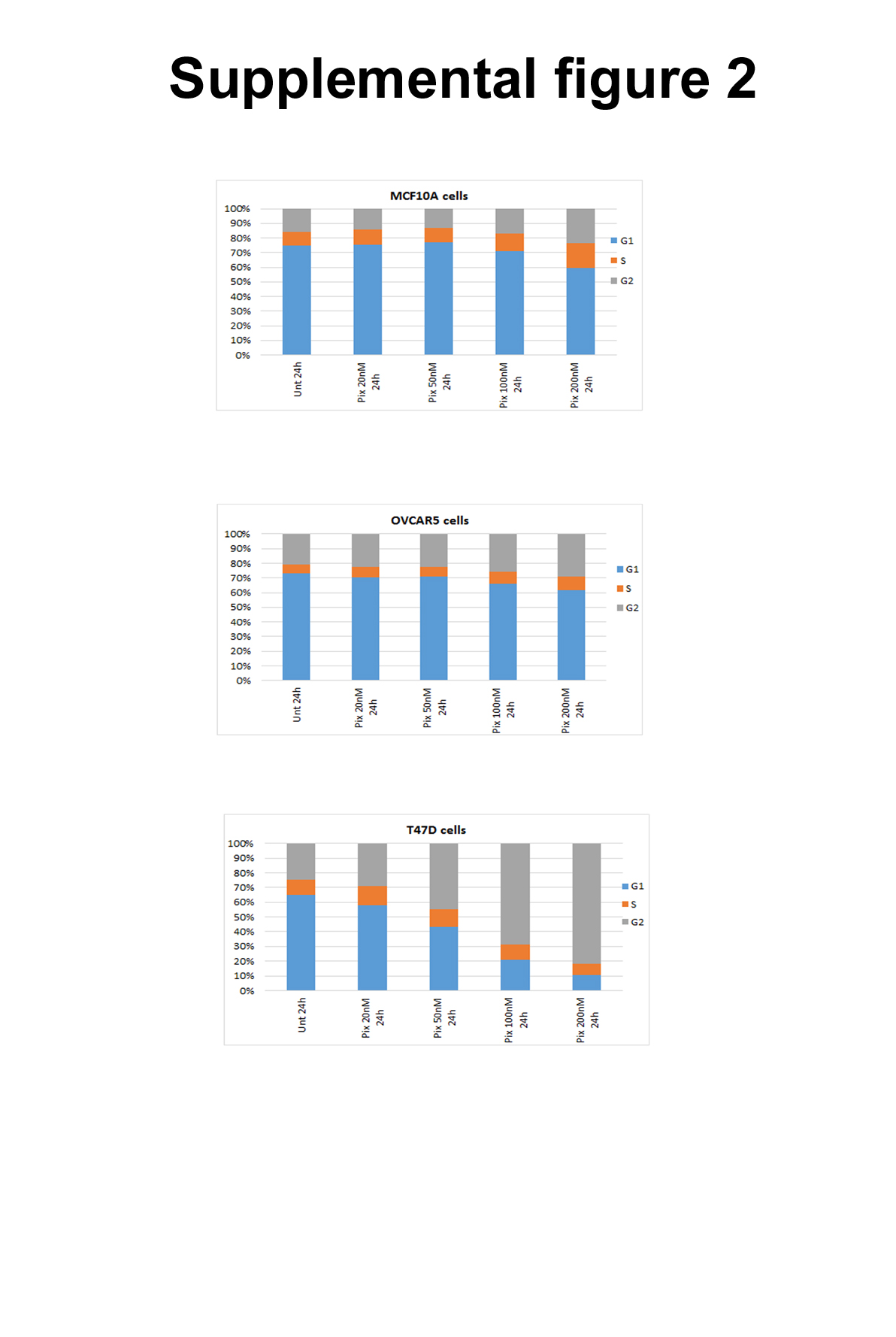

Supplement: Supplemental Figures and Captions [file kcbt-16-09-1070979-s001.zip › Figure S2.jpg]
